# Opening of the Extension of the Central Throat and Ear Hospital

**Published:** 1906-11-24

**Authors:** 


### OPENING OF THE EXTENSION OF THE CENTRAL THROAT AND EAR HOSPITAL.

The new wing of the Central Throat and Ear Hospital, Gray's Inn Road, was opened by H.R.H. Princess Louise (Duchess of Argyll) on the 19th inst. The extension consists of a building containing an operating-theatre, an electric lift, three single-bed wards, a larger ward for five beds, and a ward for the temporary reception of children undergoing minor operations, and has cost nearly £7,000. Of that sum, nearly £6,000 was collected before the opening, nad at the ceremony donations promised, and the contents of purses which H.R.H. graciously received, practically cleared off the remainder. The hospital committee have, however, prepared plans for an extension which would have doubled the present accommodation for in-patients, but have determined not to proceed with it until they have the necessary funds.

The Princess, who was accompanied by the Duke of Argyll, on arriving was conducted to a marquee draped with red and white bunting, which had been erected on the vacant ground on which the Hospital Committee hope to build their further extension. Mr. George Wallis, Chairman of Committee, then gave an account of the history of the hospital from its small inception in 1874, in a house close to the site of the present building, up to the present day. He was followed by Dr. Dundas Grant, the Senior Surgeon to the hospital, who gave a brief and interesting account of the progress of surgical science in dealing with diseases of the ear, nose, and throat during the last thirty years. After these addresses, Her Royal Highness performed what she defined as "the pleasant duty" of declaring the new wing open, and the Chaplain, the Rev. Frederick Bolingbroke, Vicar of St. Jude's, Gray's Inn Road, offered up a dedication prayer. A vote of thanks to Her Royal Highness was then moved by Mr. Albert Calkin Lewis, Vice-Chairman of Committee, and seconded by Mr. Seymour Lucas, R.A. The Duke of Argyll replied on behalf of the Princess, and expressed his hope that the hospital might soon be completed by the erection of the other wing which the Committee had planned. Mr. Philip Wilson, M.P., who moved a resolution wishing prosperity to the hospital, dwelt on the fact that the bulk of the patients came from outside the neighbourhood, and the same fact was brought forward by Mr. Carmichael Thomas, a member of Committee, in replying to the resolution, coupled with the remark that while the country patients outnumbered the London ones, they contributed very little to the hospital funds. Dr. Orwin, the consulting physician, spoke with satisfaction of the fact that many of the patients were sent to the hospital by their own medical men, thus showing the confidence which these felt in the work done there. In the course of the proceedings the Chairman received a letter from the Secretary to H.R.H. the Duke of Connaught, who is Patron of the hospital, expressing his good wishes for the success of the gathering and for the well-being of the institution, and his satisfaction at the part which his sister was taking in the opening. After the speeches the Princess received a number of purses, which were presented by ladies and children, and the contents of which formed a substantial addition to the gifts of the day.

